# Association between housing type and accelerated biological aging in different sexes: moderating effects of health behaviors

**DOI:** 10.18632/aging.203447

**Published:** 2021-08-29

**Authors:** Ted Kheng Siang Ng, David Bruce Matchar, Timothy V. Pyrkov, Peter O. Fedichev, Angelique Wei-Ming Chan, Brian Kennedy

**Affiliations:** 1 Arizona State University, Edson College of Nursing and Health Innovation, Phoenix, AZ 85004, USA; 2 Duke-National University of Singapore Medical School, Program in Health Services and Systems Research, Singapore; 3 Duke University School of Medicine, Department of Medicine (General Internal Medicine), Durham, NC 27710, USA; 4 GERO PTE. LTD., Singapore; 5 Moscow Institute of Physics and Technology, Dolgoprudny, Moscow Region 141700, Russia; 6 Duke-National University of Singapore Medical School, Center for Aging, Research and Education, Singapore; 7 National University of Singapore, Department of Sociology, Faculty of Arts and Social Sciences, Singapore; 8 National University of Singapore, Center for Healthy Longevity, Healthy Longevity Program and Department of Biochemistry, Yong Loo Lin School of Medicine, Singapore; 9 Singapore Institute of Clinical Sciences, A*STAR, Singapore; 10 National Cheng Kung University, Institute of Behavioral Medicine, College of Medicine, Taiwan

**Keywords:** health disparity, geroscience, social determinant of health, socioeconomic status, environmental factor

## Abstract

Introduction: Despite associated with multiple geriatric disorders, whether housing type, an indicator of socioeconomic status (SES) and environmental factors, is associated with accelerated biological aging is unknown. Furthermore, although individuals with low-SES have higher body mass index (BMI) and are more likely to smoke, whether BMI and smoking status moderate the association between SES and biological aging is unclear. We examined these questions in urbanized low-SES older community-dwelling adults.

Methods: First, we analyzed complete blood count data using the cox proportional hazards model and derived measures for biological age (BA) and biological age acceleration (BAA, the higher the more accelerated aging) (*N* = 376). Subsequently, BAA was regressed on housing type, controlling for covariates, including four other SES indicators. Interaction terms between housing type and BMI/smoking status were separately added to examine their moderating effects. Total sample and sex-stratified analyses were performed.

Results: There were significant differences between men and women in housing type and BAA. Compared to residents in ≥3 room public or private housing, older adults resided in 1–2 room public housing had a higher BAA. Furthermore, BMI attenuated the association between housing type and BAA. In sex-stratified analyses, the main and interaction effects were only significant in women. In men, smoking status instead aggravated the association between housing type and BAA.

Conclusion: Controlling for other SES indicators, housing type is an independent socio-environmental determinant of BA and BAA in a low-SES urbanized population. There were also sex differences in the moderating effects of health behaviors on biological aging.

## INTRODUCTION

Socioeconomic status (SES) is a well-validated social determinant of health. In particular, low SES in late life represents the accumulation of lifetime stressors, resulted from the challenges and adversities experienced throughout one’s life course. Low SES is thus associated with a wide range of adverse health outcomes, including increased inflammatory and metabolic biomarkers, age-related morbidities (including stroke, chronic kidney disease, coronary heart disease, and dementia [1–4]), increased hospital re-admissions [5–8], and increased healthcare utilization [8, 9]. Specifically, Singaporeans belonging to the lower SES strata had higher healthcare utilizations [9]. Due to the various barriers they faced, they preferred alternative medicine or self-medication to manage their health issues [10], which may exacerbate their existing conditions.

Most SES studies have focused on examining the canonical SES indicators, including educational level [11–14], income [9, 15], financial adequacy [16], and occupational class [17]. However, another SES indicator, housing type, has been understudied. Apart from indicating SES, housing type is also an indicator of environmental factors. We have previously demonstrated that environmental factors are critical social determinants of health and impact aging biomarkers and social measures [18–22]. Adopting the biopsychosocial-ecological model, specifically the Transdisciplinary Neighborhood Health Framework [23], in the present study, housing type is thus being referred to as the socio-environmental indicator or determinant of health. Housing type may predominantly determine one’s home environment and housing quality, including the characteristics of one’s indoor built environment and the surrounding areas' environments. Another closely-relevant model, the social-determinant framework further emphasizes that amongst other determinants, improving housing and living conditions are central to improving the health of urban populations [24], which could reduce morbidity, mortality, and disparities in health [24]. Conversely, low housing quality in urban neighbourhoods has previously been linked to a variety of adverse health outcomes [25–29]. Thus, individuals living in deprived areas and public rental housing have been shown to have a higher mortality rate [9, 30–32]. Relocation to temporary housing after a disaster has also been shown to suffer from negative health effects due to health behavior [33]. In Singapore, housing policy had been employed as a social engineering tool during the nation-building period to prevent the formation of ghettos [34]. Despite seemingly homogenous, emerging evidence showed that Singaporeans staying in 1–2 room public housing entailed higher healthcare utilization and had higher risks of hospital re-admission [9, 35].

These multiple lines of evidence underscore the importance of examining housing type as a key socio-environmental determinant, especially in health disparity research. However, there is a significant dearth of research on the association between housing type and health outcomes in vulnerable older adults [36]. The environmental characteristics of low-SES housing, such as the lack of recreational areas in these communities, may discourage involvement in physical activity, contributing to increased cases with obesity [16, 26]. In response to the psychosocial stressors, residents in low-SES housing could adopt unhealthy coping behaviors, such as smoking [16]. The lack of access to healthy foods in these neighbourhoods could also be another moderating factor of low-SES housing to adverse health outcomes [26]. Hence, the cumulative health disadvantage conferred by low housing type on older adults could potentially be ameliorated by adopting healthy behaviors [37].

Singapore is an affluent country, with a gross domestic product of approximately S$73,000 and a multi-ethnic population of 5.6 million residents [9]. Amongst others, home ownership is a key local indicator of SES in Singapore [9, 38]. There are two general types of housing in Singapore [34, 38]; Heavily subsidized public housing is available for citizens and permanent residents to purchase from the Housing Development Board (HDB), a government agency, on a 99-year lease, with approximately 90% of Singapore residents residing in these estates. The other 10%, who are mostly residents earning higher income/belonging to the higher SES status, reside in private housing that is landed property. Apart from the public/private housing division, it is noteworthy that in Singapore, residents staying in 1–2 room public housing were considered as staying in low-SES public housing [9, 35]. Specifically, the 1–2 room apartments in Singapore are heavily subsidized by the Singaporean government’s HDB to cater to the poor and needy citizens with no other housing options, i.e. households with a gross monthly income of below S$2,000 (~USD1589) for purchase or below S$1,500 (~1191) for rent [35]. Those residing in 1–2 room housing thus have significantly higher proportions of adverse health outcomes. Specifically, they are significantly older (based on chronological age), with a lower proportion being employed and thus significantly lower income, and have significantly worse health outcomes, including higher risks of frequent hospital admissions, readmission within 15 and 30 days, emergency department attendances, higher rates of chronic diseases, and a lower self-rated health [9, 35]. However, although low-SES housing has been demonstrated as an independent predictor of adverse health outcomes in Singapore, those studies were limited by self-reporting nature and required linkages to hospital medical records for retrieving relevant data. The former presents the issue of subjectivity and self-recall bias, while the latter poses the issue of feasibility and data confidentiality. Furthermore, whether housing type independently acts as a risk factor while accounting for other SES indicators has not been examined. Even if positive, whether housing type could exert a biological weathering effect, in which stressors cause wear and tear of the body [16, 39, 40], and thus resulting in accelerated biological aging in older adults residing in 1–2 room public housing remains unknown. Thus, examining biological aging in this specific population may resolve these issues, while present an easy-to-measure and -monitor objective indicator to inform housing and public health policies.

The Geroscience Hypothesis proposes that biological aging is a shared and significant risk factor for developing many geriatric syndromes [41, 42]. Leukocyte telomere length (LTL) and allostatic load (AL) are two examples of canonical measures of biological aging [39, 43]. Recently, several DNA-methylation-based biological age (BA) measures have been proposed, including the Horvath’s Clock [44], Hannum’s Clock [45], and Levine’s DNAm PhenoAge [46]. However, there have been several limitations to using some of these measures. Most prominently, many of these measures pre-screened candidate biomarkers against chronological age (CA) as their first step in deriving the respective BAs. Despite seeing the value in performing this procedure, the variability in aging intended to be captured by BA have been minimized [47, 48]. Furthermore, due to the complexity and costs involved in examining DNA methylation markers, the use of these BAs at the population level could be limited. Hence, a novel BA measure, utilizing mortality as the sole external criterion has been proposed recently [46, 48]. In this study, we employed the same approach and proposed a BA as a composite of complete blood count (CBC) and selected standard blood biochemistry parameters, a panel of easily accessible and examined clinical biomarkers. We have previously validated this BA model and the derived biological age acceleration (BAA) measure by showing their significant associations with multiple physical and mental health measures [49, 50]. Furthermore, we showed the BA was a dynamic indicator of organism health and was thus modifiable by health behaviors, including smoking status [49, 50].

Despite a small number of studies associating low-SES housing with adverse phenotypic outcomes, the underlying biological mechanisms are yet to be elucidated. We thus hypothesized that one of the mechanisms through which SES indicators and social-environmental factors, including housing type, impact adverse health outcome could be through the biological weathering effect on BA, resulting in accelerated biological aging. However, inconsistent findings were reported on the associations between other indicators of SES and leukocytes telomere length (LTL), including studies not finding a significant association between SES and LTL [51–53]. On the other hand, several studies elucidated significant associations between SES and DNA-methylation-derived BA. For example, Simons et al. (2016) demonstrated that low-income status was robustly associated with Hannum’s BA [16]. Despite the plethora of studies on other indicators, whether housing type is significantly associated with BA has been scarcely investigated. To our knowledge, only one study showed that homeownership was associated with a lower LTL-measured BA [51]. However, it is worth noting that not all older adults who resided in low-SES housing would experience accelerated biological aging. The exact aggravating factors further contributing to this susceptibility are unclear. Given that individuals with low-SES often have higher body mass index (BMI) and are more likely to smoke, it is thus unclear as to whether BMI and smoking status alleviate or aggravate the association between low-SES housing and BA and BAA. Motivated by a previous study [13], understanding the associations between housing type and BA, focusing on the moderating effects of health behaviors, may highlight intervention targets and approaches to ameliorating health disparities experienced by older adults residing in low-SES housing.

Aiming to address the gaps in knowledge, we conducted a study with a sample population comprising predominantly low-SES older community-dwelling Singaporean adults. This study has three aims:

1. The main aim of this study was to determine if there were significant associations between housing type and BA/BAA (indicating accelerated or decelerated aging);
2. We examined if BMI and smoking status, both as indicators of health behaviors, moderated the hypothesized associations between housing type and BA/BAA;
3. We explored the proposed differential associations and moderating effects in two subgroups, i.e. men and women.

## RESULTS

### Demographics and characteristics of BA

Table 1 summarizes the baseline characteristics of the 376 study participants. In the total sample, the mean CA was 72.20 (SD = 8.003, range = 54 to 95). The mean BA was 0.335 (SD = 0.63, range = −1.122 to 2.566) in log-odds units and 72.20 (SD = 1.48, range = 70.4 to 97.1) when scaled to years, whereas the mean BAA was 0.044 (SD = 0.563, range = –1.393 to 2.034) in log-odds units and 0.0 (SD = 1.37, range = –3.9 to 22.0) when scaled to years. Below we report BA and BAA in log-odds units. Most of the participants were women (*n* = 249, 66.2%) and Chinese (*n* = 313, 83.2%). Compared to women, there was a higher proportion of men staying in 1–2 room public housing (*n* = 108, 85% versus women, *n* = 156, 62.7%; *p* < 0.001), longest occupation held at clerical level (*n* = 25, 19.7% versus women *n* = 12, 4.8%; *p* < 0.001), having higher proportion of perceiving having difficulty in income adequacy (*n* = 58, 46.4% versus women, *n* = 87, 35.1%; *p* = 0.043), higher BA (mean = 0.451, SD = 0.573 versus women mean = 0.276, SD = 0.650, *p* = 0.008), higher proportion of smokers (*n* = 27, 21.3% versus women *n* = 8, 3.2%, *p* < 0.001), and alcohol drinkers (*n* = 29, 22.8% versus women *n* = 20, 8%, *p* < 0.001). On the other hand, there was a higher proportion of women who had no formal education (*n* = 151, 60.6% versus men *n* = 56, 44.1%, *p* = 0.003). Women also had higher mean and a wider spread of BMI values (mean = 24.900, SD = 5.086 versus men mean = 23.098, SD = 3.835; *p* < 0.001), and higher physical activity levels (mean = 1.104, SD = 0.932 versus men, mean = 0.890, SD = 0.857; *p* = 0.031). The significant differences between men and women in both housing type and BA supported our aim 3. Hence, for all regression analyses, apart from conducting total sample analyses, we also performed sex-stratified analyses.

**Table 1:** Demographic characteristics and biological age of the participants.

**Demographic and biological age variables**	**Men (*N* = 127, 33.8%) Mean ± SD or *n* (%)**	**Women (*N* = 249, 66.2%) Mean ± SD or *n* (%)**	**Total (*N* = 376, 100%) Mean ± SD or *n* (%)**	***p*-values**
**Housing type**				
1–2-room public housing	108 (85)	156 (62.7)	264 (70.2)	<0.001^***^
3-room public housing and above or private housing	19 (15)	93 (37.3)	112 (29.8)	
**Education level**				
No formal education	56 (44.1)	151 (60.6)	207 (55.1)	0.003^**^
Primary education & above	71 (55.9)	98 (39.4)	169 (44.9)	
**Longest occupational role**				
Clerical level	25 (19.7)	12 (4.8)	37 (9.8)	<0.001^***^
Executive level	102 (80.3)	237 (95.2)	339 (90.2)	
**Monthly income**				
< SGD 500	92 (76)	171 (74)	263 (74.7)	0.701
≥ SGD 500	29 (24)	60 (26)	89 (25.3)	
**Perceived income adequacy**				
Having difficulty	58 (46.4)	87 (35.1)	145 (38.9)	0.043^*^
Not much difficulty	67 (53.6)	161 (64.9)	228 (61.1)	
Biological age	0.451 ± 0.573	0.276 ± 0.650	0.335 ± 0.630	0.008^**^
Biological age acceleration	0.029 ± 0.524	0.0518 ± 0.583	0.044 ± 0.563	0.712
Chronological age	72.85 ± 7.958	71.86 ± 8.021	72.20 ± 8.003	0.259
**Ethnicity**				
Chinese	106 (83.5)	207 (83.1)	313 (83.2)	1
Other ethnicities	21 (16.5)	42 (16.9)	63 (16.8)	
**Marital Status**				
Married	65 (51.2)	143 (57.4)	208 (55.3)	0.273
Not married	62 (48.8)	106 (42.6)	168 (44.7)	
BMI	23.098 ± 3.835	24.900 ± 5.086	24.293 ± 4.774	<0.001^***^
**Smoking status**				
Smoker	27 (21.3)	8 (3.2)	35 (9.3)	<0.001^***^
Non-smoker	100 (78.7)	241 (96.8)	341 (90.7)	
**Alcohol consumption**				
Drinker	29 (22.8)	20 (8)	49 (13)	<0.001^***^
Non-drinker	98 (77.2)	229 (92)	327 (87)	
Physical activity level	0.890 ± 0.857	1.104 ± 0.932	1.032 ± 0.912	0.031^*^
Social activity level	1.236 ± 0.750	1.353 ± 0.748	1.314 ± 0.750	0.152

SGD = Singapore Dollar, 1 SGD = 0.74 USD; Biological age acceleration (BAA) was calculated by subtracting the corresponding age- and sex-standardized biological age (BA) norms from the individual BA. A positive BAA value indicated accelerated biological aging, whereas a negative value indicated decelerated biological aging. Zero value represented equal rate of biological and chronological aging. ^*^for *p* < 0.05, ^**^for *p* < 0.01, and ^***^for *p* < 0.001.

### Associations between housing type and BA/BAA

Table 2 presents the findings from the multivariate regression models, regressing BA and BAA separately on housing type, sequentially controlling for additional covariates.

**Table 2:** Associations between housing type and biological age/biological age acceleration.

	**Models**	**Biological age**			**Biological age acceleration**		
		**β (95% CI)**	***P* value**	***R^2^***	**β (95% CI)**	***P* value**	***R^2^***
**Total sample**							
Housing type	1	0.263 (0.120 to 0.406)	<0.001^***^	0.036	0.156 (0.027 to 0.285)	0.018^*^	0.016
	2	0.173 (0.024 to 0.323)	0.023^*^	0.091	0.166 (0.028 to 0.305)	0.019^*^	0.019
	3	0.199 (0.038 to 0.36)	0.015^*^	0.104	0.183 (0.033 to 0.334)	0.017^*^	0.025
	4	0.193 (0.032 to 0.354)	0.019^*^	0.121	0.180 (0.029 to 0.331)	0.020^*^	0.031
	5	0.189 (0.027 to 0.35)	0.022^*^	0.132	0.178 (0.027 to 0.330)	0.021^*^	0.046
**Men**							
Housing type	1	0.188 (−0.104 to 0.479)	0.204	0.014	0.202 (−0.064 to 0.467)	0.135	0.019
	2	0.148 (−0.144 to 0.440)	0.318	0.050	0.192 (−0.079 to 0.463)	0.163	0.021
	3	0.101 (−0.201 to 0.404)	0.508	0.120	0.154 (−0.130 to 0.438)	0.285	0.074
	4	0.109 (−0.188 to 0.405)	0.468	0.199	0.155 (−0.126 to 0.436)	0.277	0.136
	5	0.108 (−0.189 to 0.405)	0.471	0.212	0.154 (−0.127 to 0.436)	0.280	0.151
**Women**							
Housing type	1	0.246 (0.073 to 0.419)	0.006^**^	0.034	0.162 (0.006 to 0.319)	0.042^*^	0.018
	2	0.176 (−0.003 to 0.355)	0.054	0.086	0.163 (−0.004 to 0.329)	0.055	0.018
	3	0.256 (0.062 to 0.450)	0.010^*^	0.114	0.218 (0.036 to 0.400)	0.019^*^	0.034
	4	0.259 (0.065 to 0.452)	0.009^**^	0.139	0.221 (0.039 to 0.403)	0.018^*^	0.051
	5	0.249 (0.054 to 0.444)	0.013^*^	0.151	0.209 (0.026 to 0.392)	0.026^*^	0.072

^*^indicates *p* < 0.05, ^**^indicates *p* < 0.01, and ^***^indicates *p* < 0.001. Abbreviations: 95% CI: 95% confidence interval; BMI: body-mass index.

Model 1: no covariates controlled for; with 3 or more room public or private housing indicating high SES-housing, which was the reference group in all regression models.

Model 2: added chronological age, sex, ethnicity.

Model 3: added marital status, education level, longest occupational role, income level, perceived income adequacy.

Model 4: added BMI, smoking status, alcohol consumption.

Model 5: added physical and social activity levels.

### Total sample

**Figure 1:**
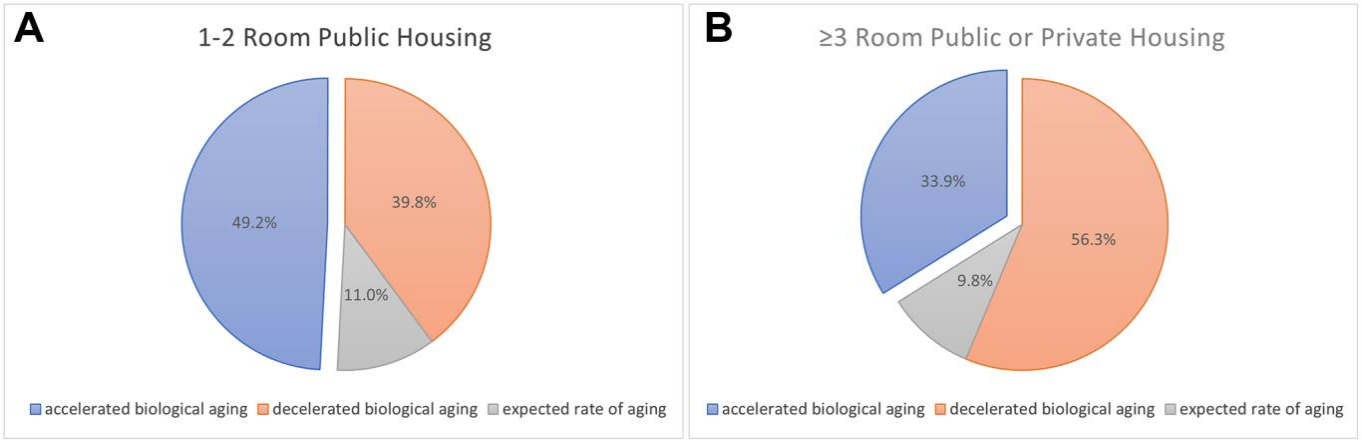
(**A**) The number and proportions of older adults resided in 1–2 room public housing who had accelerated biological aging was higher (*N* = 130, 49.2%), compared to those with decelerated biological aging (*N* = 105, 39.8%). There were 29 older adults with equal rate of biological aging with chronological aging (11%). (**B**) The number and proportions of older adults resided in 3 room or higher public housing or private housing who had accelerated biological aging was lower (*N* = 38, 33.9%), compared to those with decelerated biological aging (*N* = 63, 56.3%). There were 11 older adults with equal rate of biological aging with chronological aging (9.8%).

The participants who resided in 1–2 room public housing had higher BA than those residing in 3 or more room public housing or private housing, even upon fully controlling for covariates (model 5: β = 0.189, 95% CI = 0.027 to 0.350, *p* = 0.022). Similarly, there was a significant association between housing type and BAA, with the participants residing in 1–2 room public housing had accelerated biological aging (model 1: β = 0.156, 95% CI = 0.027 to 0.285, *p* = 0.018). The relationship remained significant upon fully controlling for all the covariates (model 5: β = 0.178, 95% CI = 0.027 to 0.330, *p* = 0.021). Notably, for all the other SES indicators, although some were significant in the bivariate associations (Supplementary Table 1A, 1B and 1C), after controlling for each other and housing type, none were significantly associated with both BA and BAA (Supplementary Table 2A). Additional descriptive analyses can be found in the Supplementary Materials and Figure 1A and 1B.

### Sex-stratified analyses

Sex-stratified analyses showed similar results in women for both BA and BAA, upon controlling for all covariates (BA, left panel, model 5: β = 0.249, 95% CI = 0.054 to 0.444, *p* = 0.013; BAA, right panel, model 5: β = 0.209, 95% CI = 0.026 to 0.392, *p* = 0.026). However, in men, no significant associations were found between housing type and either BA or BAA. In men, the standardized β for the associations between housing type and BMI with BAA were 0.105 and 0.242, respectively (Supplementary Table 2B). While in women, for the association with BAA, the standardized β for the associations between housing type and BMI with BAA were 0.173 and < 0.001, respectively (Supplementary Table 2C).

### Moderation by health behaviors

#### Total sample analyses

In the total sample, there was a significant interaction effect between housing type and BMI on both BA (β = −0.041, 95% CI = −0.071 to −0.011, *p* = 0.007) and BAA (β = −0.030, 95% CI = −0.058 to −0.002, *p* = 0.039) (Table 3). However, no significant interaction effect was observed between housing type and smoking status on either BA (β = 0.191, 95% CI = −0.430 to 0.812, *p* = 0.545) or BAA (β = 0.210, 95% CI = −0.372 to 0.792, *p* = 0.478) (Table 3).

**Table 3:** Associations between housing type and biological age/biological age acceleration, models with the additions of interaction terms for health behaviors.

	**Models**	**Biological age**			**Biological age acceleration**		
		**β (95% CI)**	***P*-value**	***R^2^***	**β (95% CI)**	***P*-value**	***R^2^***
**Total sample**							
Housing Type	1	0.228 (0.067 to 0.388)	0.006^**^	0.114	0.178 (0.027 to 0.330)	0.021^*^	0.046
Housing Type	2	−0.183 (−1.402 to 1.036)	0.768	0.133	−0.231 (−1.373 to 0.912)	0.691	0.047
Smoking Status		0.104 (−0.474 to 0.682)	0.723		0.163 (−0.378 to 0.705)	0.553	
Housing Type x Smoking Status		0.191 (−0.430 to 0.812)	0.545		0.210 (−0.372 to 0.792)	0.478	
Housing Type	3	1.178 (0.438 to 1.918)	0.002^**^	0.150	0.896 (0.199 to 1.593)	0.012^*^	0.058
BMI		0.045 (0.020 to 0.071)	0.001^**^		0.030 (0.006 to 0.054)	0.015^*^	
Housing Type x BMI		−0.041 (−0.071 to −0.011)	0.007^**^		−0.030 (−0.058 to −0.002)	0.039^*^	
**Men**							
Housing Type	1	0.108 (−0.189 to 0.405)	0.471	0.212	0.154 (−0.127 to 0.436)	0.280	0.151
Housing Type	2	−1.363 (−2.761 to 0.034)	0.056	0.245	−1.259 (−2.584 to 0.066)	0.062	0.187
Smoking Status		0.611 (−0.092 to 1.314)	0.088		0.634 (−0.032 to 1.301)	0.062	
Housing Type x Smoking Status		0.795 (0.057 to 1.534)	0.035^*^		0.764 (0.063 to 1.464)	0.033^*^	
Housing Type	3	0.213 (−1.813 to 2.239)	0.835	0.212	0.267 (−1.654 to 2.188)	0.783	0.151
BMI		0.043 (−0.040 to 0.127)	0.302		0.037 (−0.041 to 0.116)	0.348	
Housing Type x BMI		−0.005 (−0.092 to 0.082)	0.918		−0.005 (−0.087 to 0.078)	0.907	
**Women**							
Housing Type	1	0.249 (0.054 to 0.444)	0.013^*^	0.151	0.209 (0.026 to 0.392)	0.026^*^	0.072
Housing Type	2	1.556 (−0.651 to 3.763)	0.166	0.157	1.397 (−0.674 to 3.468)	0.185	0.077
Smoking Status		−0.214 (−1.177 to 0.749)	0.662		−0.115 (−1.019 to 0.788)	0.802	
Housing Type x Smoking Status		−0.665 (−1.783 to 0.453)	0.243		−0.604 (−1.654 to 0.445)	0.257	
Housing Type	3	1.381 (0.521 to 2.240)	0.002^**^	0.179	0.982 (0.170 to 1.795)	0.018^*^	0.087
BMI		0.037 (0.009 to 0.065)	0.009^**^		0.020 (−0.006 to 0.046)	0.137	
Housing Type x BMI		−0.046 (−0.081 to −0.012)	0.008^**^		−0.032 (−0.064 to 0.001)	0.056	

^*^indicates *p* < 0.05, ^**^indicates *p* < 0.01. Abbreviations: 95% CI: 95% confidence interval; BMI: body-mass index.

Model 1: added chronological age, sex, ethnicity, marital status, education level, longest occupational role, income level, perceived income adequacy, BMI, smoking status, alcohol consumption, physical and social activity levels (respective models 5 from Table 2).

Model 2: separately added interaction term: Housing^*^Smoking Status.

Model 3: separately added interaction term: Housing^*^BMI.

#### Sex-stratified analyses

In women, as with the total sample, the interaction effect between housing type and BMI was significant (Table 3). Women resided in 1–2 room public housing and who had higher BMI had a lower BA (β = −0.046, 95% CI = −0.081 to −0.012, *p* = 0.008) and lower BAA (β = −0.032, 95% CI = 0.064 to 0.001, *p* = 0.056), compared to those resided in 3 or more room public housing or private housing and with lower BMI. No significant interaction effect between housing type and smoking status was detected in women.

On the other hand, we observed the opposite trends in men; Although no significant interaction effect between housing type and BMI was found, we observed a statistically significant interaction effect between housing type and smoking status (Table 3). Men resided in 1–2 room public housing and who were smokers had a higher BA (β = 0.795, 95% CI = 0.057 to 1.534, *p* = 0.035) and higher BAA (β = 0.764, 95% CI = 0.063 to 1.464, *p* = 0.033), indicating accelerated biological aging.

#### Additional analyses

Despite not being employed as an external criterion, chronological age was significantly associated with BA (β = 3.133, *p* ≤ 0.001). Furthermore, there were both a higher number and proportion of participants (*p* = 0.011) staying in 1–2 room public housing who had accelerated biological aging (*N* = 130, 49.2%), compared to the reference (*N* = 29, 11%) and decelerated aging (*N* = 105, 39.8%) (Figure 1A). Whereas for participants staying in 3 room or higher public housing or private housings (Figure 1B), there were both a lower number and proportion of participants who had significantly accelerated aging (*N* = 38, 33.9%), compared to participants with no change/the reference (*N* = 11, 9.8%) and decelerated aging (*N* = 63, 56.3%).

## DISCUSSION

Controlling for a comprehensive panel of SES indicators, in the total sample, as compared to the high-SES housing type, we showed pilot data on the independent associations between low-SES housing and advanced BA and BAA. In sex-stratified subgroup analyses, the association was only observed in women, suggesting sex-specific vulnerability of women to the weathering effect of housing type as a socio-environmental determinant on biological aging. Furthermore, examining health behaviors as the hypothesized moderators, we showed sex-specific and differential moderating effects of BMI and smoking status on the association between housing type and biological aging. In women, higher BMI was protective for those resided in 1–2 room public housing, having moderated lower BA and BAA. In men, although the main effect of housing type was not significant, smoking status moderated the association between housing type and accelerated BAA; Men resided in 1–2 room public housing and who were smokers had both higher BA and accelerated BAA. Hence, all three hypotheses were supported by the data. Taken together, we showed pilot data on the associations between housing type, a social-environmental determinant of health, and accelerated biological aging. These findings underscore the importance of examining socio-environmental and behavioral factors in a predominantly biomedical landscape in the geroscience field.

In this study, we showed two lines of evidence supportive of the instrumental roles of housing type in biological aging. First, in the descriptive analyses, we found a higher proportion of participants resided in 1–2 room public housing who had accelerated biological aging (56.3%), compared to 39.8% of participants resided in 3 or more room public housing or private housing who had accelerated biological aging. Second, performing regression analyses, further controlling for covariates, the participants resided in 1–2 room public housing were associated with higher BA and accelerated biological aging. In contrary to this study, previous studies did not comprehensively examine the associations between SES indicators/socio-environmental determinant and biological aging, having frequently overlooked housing type as an indicator. Controlling for these SES indicators as covariates are essential, since they had been shown to confound the association between SES and BAA [13, 14]. In this study, the association between housing type and BAA remained significant upon comprehensively controlled for all the other SES indicators and covariates, including demographics, health behaviors, and social and physical activities.

The nature of the SES indicators could be the discerning factor in the association and lack thereof with biological aging. Our findings are in line with the extant literature showing that house ownership, but not the other SES indicators, was significantly associated with LTL-defined BA [51]. Housing type is more than an SES indicator, as it also captures multiple facets of the environment in which the older adults live in, thus we termed it the “socio-environmental indicator” in this study. Hence, we suggest that compared to the collective influences of SES *and* environmental factors encompassed by low housing type, the other four SES indicators had comparatively lower influences on biological aging. There are several plausible mechanistic explanations; Residing in low-SES public housing could have facilitated the accumulation of life-long stressors, exposing older adults to a health-deleterious environment, culminating in a lifetime of biological weathering effects [54] and thus premature aging [54, 55]. Among the health-deleterious environments in low-SES housing could be poor insulation, poor combustion appliances, cockroach, dust mites, and rodent infestations, hyper- and hypothermia, unaffordable rent, and dangerous levels of lead in soil and household paint [56]. Indeed, biological weathering is particularly prevalent in disadvantaged sample population in an urbanized population such as ours. Similarly, another study showed that female African-Americans residing in the low-SES neighbourhood experienced excess biological weathering [40]. One limitation is that these studies did not comprehensively controlled for other SES indicators. Extending previous findings, we found significant independent association between housing type and BA. SES-associated social stressors have also been shown to independently disrupt homeostasis [57]. Amongst the validated biological pathways mediating the effects of social stressors on adverse health outcomes include disrupting the hypothalamic-pituitary-adrenal (HPA) axis, autonomic nervous system, metabolic, and immune system [39], many of which were captured in our novel BA and BAA measures.

Our sex-stratified subgroup analyses revealed that only women, but not men, resided in 1–2 room public housing experienced accelerated biological aging. This finding concurs with previous observation showing that women *and* individuals in the lower social classes are more susceptible to the detrimental effects of stressors. Apart from the differential distributions of life stressors in different social classes, the psychological and social resources to cope with stressors are also unequally accessible to different sexes. For example, for women residing in high-SES housing, possibly due to the availability of and/or socially acceptable use of residential public spaces by women in these contexts [58, 59], the effects of sex-specific stressors could be ameliorated. Conversely, for women in low-SES housing, given the unique challenges they are faced with, including marital conflicts, juggling a full-time job and the roles as a wife [60, 61], women may have less coping resources available which could be called upon in response to stressors [62]. Hence, these unique psychosocial challenges faced by women could have further compounded and reinforced the biological weathering effects exerted by low social classes, resulting in women-specific health vulnerability and disparity [62], and thus culminating in accelerated biological aging.

Lastly, exploring the moderating effects of health behaviors on the associations, in women, higher BMI counteracted the biological weathering effect of low-SES housing on biological aging. This finding is in line with the extant literature showing that in late life, in contrary to being detrimental to health, higher BMI is protective and predictive of better health [63]. Compared to their lower BMI counterparts, older adults who were obese also had lower mortality risk [57]. Conversely, epidemiological studies have shown that lower BMI could be a sign of frailty in late life [64]. According to the Asian-specific BMI standards, obesity cut-offs for women and men are 25 kg/m^2^ and 27 kg/m^2^, respectively [59]. In our study, women, but not men, were borderline obese (24.9 kg/m^2^, compared to men, 23.10 kg/m^2^), further supportive of the specific moderating effect of higher BMI on lower BAA in women. On the other hand, men who resided in 1–2 room public housing who were also smokers had significantly accelerated biological aging. This finding supports the extant literature showing that low-SES individuals may be more susceptible to engage in unhealthy stress-reducing behaviors, such as smoking [13, 16], leading to accelerated biological aging [48, 50]. Due to the low percentage (3.2%) of women who smoked, compared to 21.3% of men who were smokers (*p* < 0.001), future studies comprising more balanced smoker percentages between men and women are warranted to further examine the moderating effects of smoking status. Furthermore, our previous non-pharmacological trial showed that education level, an SES indicator, moderated the protective effect of the intervention on biomarker in women but not men [65], equally highlighting the importance of intersectionality among sex, social determinants of health, and biomarker.

There were several limitations in this study. The main limitation was the cross-sectional nature of the study, rendering us unable to determine the causal effects of housing type on BAA, leaving the possibility of reverse causation. However, an alternative interpretation of older adults who had accelerated biological aging choosing to reside in low-SES housing seems rather implausible. This study also had a relatively moderate sample size, which could have limited the value of the sex-stratified exploratory analyses on health behaviors. Hence, the absence of statistical significance in some of the sex-stratified analyses does not imply the absence of a relationship [66], warranting future studies with larger sample size and more balanced variables. Furthermore, not having a nationally-representative sample, our findings warrant replication to be generalizable to the general population. Lastly, older adults staying in 1–2 room public housing have a greater risk of developing adverse health outcomes and thus have a higher mortality rate. This issue may present competing risk of death, with participants having a higher BA could have passed away before being recruited in this study. This issue could have potentially resulted in the underestimations of the detected associations and the effect sizes. If that was the case, housing type and health behaviors could have had even greater associations and moderating effects with BAA than presented.

This study extended our understanding of the associations between housing type and biological aging on several fronts. First, concurrently examining a comprehensive profile of SES indicators, we discerned the biological weathering effects of five SES indicators, showing the independent association between housing type and BAA. Second, with the associations established, we investigated the differential moderating effects of health behaviors on the associations between housing type and BAA in different sexes, contributing pilot data to untangling the complex intersections amongst multiple social and behavioral determinants of biological aging. Third, the hypotheses were tested using a novel measure of BA, which we previously derived based on CBC and selected standard blood biochemistry parameters, overcoming several limitations present in the current operationalizations of BA. Fourth, low-SES communities are more vulnerable to social stressors and have higher rates of adverse health outcomes [9, 28], all of which could be underpinned by biological aging [34, 35]. Given our study participants were recruited from predominantly low-SES communities in Singapore, our sample population is thus well-suited to test the proposed hypotheses.

To our knowledge, this is the first study showing pilot data on an independent and significant association between housing type, an indicator of socio-environmental determinant, and biological aging, which persisted after controlling for a comprehensive panel of four SES indicators. We also showed pilot exploratory data on two specific socio-behavioural determinants through which health behaviors (BMI and smoking status) moderated accelerated biological aging in low-SES older adults. The differential moderating effects of health behaviors on these associations specific to men and women suggest sex-specific vulnerabilities of BA to social-behavioural determinants of health. The model implies that dependent on the sex, by modifying one of the risk factors/health behaviors (i.e. BMI or smoking) in older adults residing in low-SES housing, biological aging could potentially be forestalled in this group of the vulnerable population. According to the Geroscience Hypothesis, accelerated biological aging predisposes these older adults to developing a range of geriatric syndromes. Furthermore, the blood test parameters employed in this study in constructing BA are readily available for testing in commercial laboratories and routinely examined in the clinical settings. The ease of data availability, not limited by sophisticated field specimen collection procedures and laboratory examinations unlike DNA-methylation-based measures, make this BA attractive to be used at the population level. We have also previously shown that several of the BA biomarkers could be improved by non-pharmacological interventions [20, 67, 68]. Furthermore, data gathered from consumer-grade mobile and wearable device or sensor may prove as a useful tool to enroll even larger cohorts in studies of aging, as our recent work showed notable concordance between BA in blood test and wearable sensor data [50, 69, 70]. Taken together, the identifications of social and behavioral determinants of health and their associations with accelerated biological aging could highlight novel targets and approaches to preventing and closing the gaps of health disparity in low-SES communities, with the potential to inform and positively impact policies and interventions. A future direction is to validate these findings in longitudinal studies with a nationally-representative sample, especially to pinpoint the effects of specific environmental characteristics of housing types on biological aging. Despite the unique private/public divide and housing type present in Singapore, with the increasing aging population coupled with limited housing availabilities and homelessness issue worldwide, upon further validation in other countries, the findings of this study have potential implications in informing housing and public policies in other countries. Despite different cut-offs and criteria used for defining low-SES housing, studies from many other countries [25–28, 33, 36, 71] similarly found housing as a prominent social determinant of health and associated with multiple adverse health outcomes. Apart from moderating effects, whether BMI and/or smoking status also mediate the effects of low-SES housing on biological aging warrant future mediational analyses employing longitudinal cohort studies, validating intervention targets to combat health disparities in biological aging and geroscience.

## METHODS

### Study participants

The participants were recruited from the community for a behavioral randomized controlled trial (RCT) entitled Self-Care for Older PErsons (SCOPE) intervention [68, 72, 73]. This RCT was approved by the National University of Singapore Institutional Review Board (NUS-IRB Reference No: 11-111) and registered at ClinicalTrials.gov (https://www.clinicaltrials.gov/ct2/show/NCT01672177?term = scopeandcntry = SGandrank = 2). Informed consent was obtained before screening for eligible participants. In this study, we sought to analyze the data only from the baseline to avoid intervention effects on the biomarkers.

### Independent variable: housing type

Housing type was categorized into staying in: a) 1–2-room public housing (low SES housing) and b) ≥3 room public or private housing (high SES housing, served as the reference group in all regression models).

### Dependent variable: BA and BAA

#### Blood collection, plasma processing and biomarker measurements

Blood samples were collected between 08:00 and 09:00 in the morning to minimize diurnal variations. We asked the participants to stop consuming foods starting from 10 pm the night before the blood draw. They were advised to only consume plain water. Research nurses obtained blood samples via performing venipuncture. Samples were kept at 4°C for a maximum of three hours before they were processed. Whole blood samples were centrifuged at 1650 g for 25 minutes at 4^o^C to obtain the plasma. After the completion of sample collections, they were sent to the respective laboratories for measurements. The samples were assayed for CBC and other biochemical markers on the same day to avoid the batch effect. Biomarkers were examined using commercially available assay kits and procedures as per the instructions of the respective manufacturers of the kits and laboratories.

### Covariates: other SES indicators

Education level was operationalized as two groups, no formal education and with primary school education and above. The longest occupation held was operationalized as two groups: 1) clerical level, comprising clerks, sales and production, cleaners/labourers, and housemakers, 2) executive level, comprising professional, managerial levels, and associates. Monthly income was divided into < SGD 500 and ≥ SGD 500 (1 SGD = 0.74 USD). Lastly, perceived income adequacy was operationalized as two groups: 1) having difficulty, including participants who reported having some difficulty and having much difficulty paying expenses, 2) having not much difficulty, combining participants who reported having enough and just enough money to spend.

Detailed descriptions for other covariates can be found in the Supplementary Materials.

### Moderators: health behaviors

BMI was defined as weight divided by squared height (unit = kg/m^2^). Smoking Status was determined by asking whether the participants were current smokers.

### Statistical analyses

#### Sample size calculation

Based on a power calculation with 80% power at 5% significance with a 2-tailed test, a sample size of 65 could detect an effect size of 0.35 for a significant correlation. Hence, the targeted total sample size needed to be 65 or more.

### Biological age (BA)

The biological age (BA) model was trained using the UK Biobank data. Following [48, 74], we characterized UK Biobank participants with a binary label as disease-free if they were not diagnosed as having any of the following health conditions: cancer (C00–C99), diabetes (E10–E14), hypertension (I10–I15), Ischaemic heart diseases (I20–I25), CHF (I50), stroke (I60–I64), emphysema (J43, J44), arthritis (M00–M25), as well as self-reported data. We used the binary label for logistic regression which is an approximation to the proportional hazards model to train the model. The approximation works particularly well when the disease rate is small [75, 76] which is the case in the dataset (19% of the studied population). Analyses were performed using the sklearn (version 0.20.3) and lifelines (version 0.19.5) packages in python.

The total sample of UK Biobank (214517 female, 186480 male) was split into training (80%) and test sets. Following our previous method [50], we used CBC parameters to produce an organism state indicator. We also used selected parameters from the standard biochemistry profile to produce more robust associations with health status. The complete list of markers include: Hemoglobin (g/dL), Red blood cell count (million cells/uL), Mean cell volume (fL), Mean Cell Hgb Conc. (g/dL), Red cell distribution width (%), Platelet count (1000 cells/uL), Lymphocyte number (1000 cells/uL), Monocyte number (1000 cells/uL), Neutrophils num (1000 cell/uL), Eosinophils number (1000 cells/uL), Basophils number (1000 cells/uL), Albumin (g/L), Creatinine (umol/L), Cholesterol (mmol/L), HDL-Cholesterol (mmol/L), LDL-cholesterol (mmol/L), Triglycerides (mmol/L). The model yielded ROC AUC for the binary label of 0.66 for both the training and test sets and concordance index for mortality follow-up of 0.64 and 0.65 for the training and test sets, respectively, which was consistent with what was reported for CBC only in our previous study [50].

Finally, we converted the predicted log-odds ratio to biological age using scaling and offset following the approach outlined previously [46]. For this study which focused on the low-SES population, we used Singapore's SCOPE dataset as the reference for scaling, since UK Biobank might represent an enrollment bias for such population [77].

### Biological age acceleration (BAA)

BAA, an indicator for being biologically older or younger, was calculated by subtracting the BA from the corresponding average BA for age- and sex-matched cohorts. A positive BAA value indicated accelerated aging, whereas a negative value indicated decelerated aging. Zero represented one has the expected BA based on one’s CA.

### Regression analyses

To address aim 1, we performed linear regression analyses associating housing type (independent variable) with i) BA and ii) BAA (both as dependent variables) in separate models. In the multivariate regression analyses, all the models were conceptualized and co-variates selected *a priori*, with additional groups of covariates sequentially entered into the regression models. Model 1 did not control for any covariates, solely examining the bivariate relationships. Model 2 controlled for chronological age (CA), sex, and ethnicity. Model 3 further controlled for marital status and four other SES indicators, namely education level, the longest occupation held, income level, and perceived income adequacy. Model 4 further controlled for BMI, smoking status, and alcohol drinker. Lastly, model 5 further controlled for social and physical activities.

To address aim 2, we examined the hypothesized moderating effects of BMI and smoking status separately, by adding interaction terms between the variables with housing type in separate regression models, on top of the models examined in aim 1.

Lastly, to address aim 3, we repeated the same analyses delineated above, the only difference was that instead of the total sample, we performed sex-stratified analyses.

All the regression analyses were performed using Statistical Package for the Social Sciences (SPSS) version 24.0 (IBM SPSS Statistics for Windows, Version 24.0). A two-tailed *p*-value of < 0.05 was considered statistically significant.

## Supplementary Materials

Supplementary Materials

Supplementary Tables

## References

[1] Gruenewald TL, Cohen S, Matthews KA, Tracy R, Seeman TE. Association of socioeconomic status with inflammation markers in black and white men and women in the Coronary Artery Risk Development in Young Adults (CARDIA) study. Soc Sci Med. 2009; 69:451–59. 10.1016/j.socscimed.2009.05.018 19524346 PMC2747365

[2] Hemingway H, Shipley M, Britton A, Page M, Macfarlane P, Marmot M. Prognosis of angina with and without a diagnosis: 11 year follow up in the Whitehall II prospective cohort study. BMJ. 2003; 327:895. 10.1136/bmj.327.7420.895 14563744 PMC218810

[3] Koster A, Bosma H, Penninx BW, Newman AB, Harris TB, van Eijk JT, Kempen GI, Simonsick EM, Johnson KC, Rooks RN, Ayonayon HN, Rubin SM, Kritchevsky SB, and Health ABC Study. Association of inflammatory markers with socioeconomic status. J Gerontol A Biol Sci Med Sci. 2006; 61:284–90. 10.1093/gerona/61.3.284 16567379

[4] Loucks EB, Pilote L, Lynch JW, Richard H, Almeida ND, Benjamin EJ, Murabito JM. Life course socioeconomic position is associated with inflammatory markers: the Framingham Offspring Study. Soc Sci Med. 2010; 71:187–95. 10.1016/j.socscimed.2010.03.012 20430502 PMC2895737

[5] Krumholz HM, Bernheim SM. Considering the role of socioeconomic status in hospital outcomes measures. Ann Intern Med. 2014; 161:833–34. 10.7326/M14-2308 25437411 PMC5459391

[6] Arbaje AI, Wolff JL, Yu Q, Powe NR, Anderson GF, Boult C. Postdischarge environmental and socioeconomic factors and the likelihood of early hospital readmission among community-dwelling Medicare beneficiaries. Gerontologist. 2008; 48:495–504. 10.1093/geront/48.4.495 18728299

[7] Hu J, Gonsahn MD, Nerenz DR. Socioeconomic status and readmissions: evidence from an urban teaching hospital. Health Aff (Millwood). 2014; 33:778–85. 10.1377/hlthaff.2013.0816 24799574

[8] Filc D, Davidovich N, Novack L, Balicer RD. Is socioeconomic status associated with utilization of health care services in a single-payer universal health care system? Int J Equity Health. 2014; 13:115. 10.1186/s12939-014-0115-1 25431139 PMC4260253

[9] Low LL, Wah W, Ng MJ, Tan SY, Liu N, Lee KH. Housing as a Social Determinant of Health in Singapore and Its Association with Readmission Risk and Increased Utilization of Hospital Services. Front Public Health. 2016; 4:109. 10.3389/fpubh.2016.00109 27303662 PMC4884736

[10] Wee LE, Lim LY, Shen T, Lee EY, Chia YH, Tan AY, Koh GC. Choice of primary health care source in an urbanized low-income community in Singapore: a mixed-methods study. Fam Pract. 2014; 31:81–91. 10.1093/fampra/cmt064 24253204

[11] Adler N, Pantell MS, O'Donovan A, Blackburn E, Cawthon R, Koster A, Opresko P, Newman A, Harris TB, Epel E. Educational attainment and late life telomere length in the Health, Aging and Body Composition Study. Brain Behav Immun. 2013; 27:15–21. 10.1016/j.bbi.2012.08.014 22981835 PMC3543785

[12] Guo N, Zhou Y, Wang T, Lin M, Chen J, Zhang Z, Zhong X, Lu Y, Yang Q, Xu D, Gao J, Han M. Specifically Eliminating Tumor-Associated Macrophages with an Extra- and Intracellular Stepwise-Responsive Nanocarrier for Inhibiting Metastasis. ACS Appl Mater Interfaces. 2020; 12:57798–809. 10.1021/acsami.0c19301 33325679

[13] Needham BL, Adler N, Gregorich S, Rehkopf D, Lin J, Blackburn EH, Epel ES. Socioeconomic status, health behavior, and leukocyte telomere length in the National Health and Nutrition Examination Survey, 1999-2002. Soc Sci Med. 2013; 85:1–8. 10.1016/j.socscimed.2013.02.023 23540359 PMC3666871

[14] Steptoe A, Hamer M, Butcher L, Lin J, Brydon L, Kivimäki M, Marmot M, Blackburn E, Erusalimsky JD. Educational attainment but not measures of current socioeconomic circumstances are associated with leukocyte telomere length in healthy older men and women. Brain Behav Immun. 2011; 25:1292–98. 10.1016/j.bbi.2011.04.010 21536122

[15] Lindenauer PK, Lagu T, Rothberg MB, Avrunin J, Pekow PS, Wang Y, Krumholz HM. Income inequality and 30 day outcomes after acute myocardial infarction, heart failure, and pneumonia: retrospective cohort study. BMJ. 2013; 346:f521. 10.1136/bmj.f521 23412830 PMC3573180

[16] Simons RL, Lei MK, Beach SR, Philibert RA, Cutrona CE, Gibbons FX, Barr A. Economic hardship and biological weathering: The epigenetics of aging in a U.S. sample of black women. Soc Sci Med. 2016; 150:192–200. 10.1016/j.socscimed.2015.12.001 26765221 PMC4733569

[17] Cherkas LF, Aviv A, Valdes AM, Hunkin JL, Gardner JP, Surdulescu GL, Kimura M, Spector TD. The effects of social status on biological aging as measured by white-blood-cell telomere length. Aging Cell. 2006; 5:361–65. 10.1111/j.1474-9726.2006.00222.x 16856882

[18] Chan HY, Ho RC, Mahendran R, Ng KS, Tam WW, Rawtaer I, Tan CH, Larbi A, Feng L, Sia A, Ng MK, Gan GL, Kua EH. Effects of horticultural therapy on elderly' health: protocol of a randomized controlled trial. BMC Geriatr. 2017; 17:192. 10.1186/s12877-017-0588-z 28851276 PMC5576101

[19] Ng KST, Sia A, Ng MKW, Tan CTY, Chan HY, Tan CH, Rawtaer I, Feng L, Mahendran R, Larbi A, Kua EH, Ho RCM. Effects of Horticultural Therapy on Asian Older Adults: A Randomized Controlled Trial. Int J Environ Res Public Health. 2018; 15:1705. 10.3390/ijerph15081705 30096932 PMC6121514

[20] Wong GCL, Ng TKS, Lee JL, Lim PY, Chua SKJ, Tan C, Chua M, Tan J, Lee S, Sia A, Ng MKW, Mahendran R, Kua EH, et al. Horticultural Therapy Reduces Biomarkers of Immunosenescence and Inflammaging in Community-Dwelling Older Adults: A Feasibility Pilot Randomized Controlled Trial. J Gerontol A Biol Sci Med Sci. 2021; 76:307–17. 10.1093/gerona/glaa271 33070170 PMC7812436

[21] Sia A, Ng KST, Ng MK, Chan HY, Tan CH, Rawtaer I, Feng L, Mahendran R, Kua EH, Ho RC. The effect of therapeutic horticulture on the psychological wellbeing of elderly in Singapore: A randomised controlled trial. Journal of Therapeutic Horticulture. 2018; 28:1–10.

[22] Ng TKS, Gan DRY, Mahendran R, Kua EH, Ho RC. Social connectedness as a mediator for horticultural therapy's biological effect on community-dwelling older adults: Secondary analyses of a randomized controlled trial. Soc Sci Med. 2021; 284:114191. 10.1016/j.socscimed.2021.114191 34271401

[23] Gan DRY. Neighborhood effects for aging in place: A transdisciplinary framework toward health-promoting settings. Hous Soc. 2017; 44:79–113. 10.1080/08882746.2017.1393283

[24] Vlahov D, Freudenberg N, Proietti F, Ompad D, Quinn A, Nandi V, Galea S. Urban as a determinant of health. J Urban Health. 2007 (3 Suppl); 84:i16–26. 10.1007/s11524-007-9169-3 17356903 PMC1891649

[25] Bashir SA. Home is where the harm is: inadequate housing as a public health crisis. Am J Public Health. 2002; 92:733–38. 10.2105/ajph.92.5.733 11988437 PMC3222229

[26] Hood E. Dwelling disparities: how poor housing leads to poor health. Environ Health Perspect. 2005; 113:A310–17. 10.1289/ehp.113-a310 15866753 PMC1257572

[27] Rauh VA, Landrigan PJ, Claudio L. Housing and health: intersection of poverty and environmental exposures. Ann N Y Acad Sci. 2008; 1136:276–88. 10.1196/annals.1425.032 18579887

[28] Fullilove MT, Fullilove RE 3rd. What's housing got to do with it? Am J Public Health. 2000; 90:183–84. 10.2105/ajph.90.2.183 10667175 PMC1446157

[29] Srinivasan S, O'Fallon LR, Dearry A. Creating healthy communities, healthy homes, healthy people: initiating a research agenda on the built environment and public health. Am J Public Health. 2003; 93:1446–50. 10.2105/ajph.93.9.1446 12948961 PMC1447991

[30] Sloggett A, Joshi H. Higher mortality in deprived areas: community or personal disadvantage? BMJ. 1994; 309:1470–74. 10.1136/bmj.309.6967.1470 7804047 PMC2541648

[31] Bowen EA, Mitchell CG. Housing as a Social Determinant of Health: Exploring the Relationship between Rent Burden and Risk Behaviors for Single Room Occupancy Building Residents. Soc Work Public Health. 2016; 31:387–97. 10.1080/19371918.2015.1137518 27167535

[32] Seng JJB, Kwan YH, Goh H, Thumboo J, Low LL. Public rental housing and its association with mortality - a retrospective, cohort study. BMC Public Health. 2018; 18:665. 10.1186/s12889-018-5583-6 29843652 PMC5975624

[33] Murakami A, Sugawara Y, Tomata Y, Sugiyama K, Kaiho Y, Tanji F, Tsuji I. Association between housing type and γ-GTP increase after the Great East Japan Earthquake. Soc Sci Med. 2017; 189:76–85. 10.1016/j.socscimed.2017.07.020 28787629

[34] Phang SY. The Singapore Model of Housing and the Welfare State. Research Collection School Of Economics. Housing and the New Welfare State: Perspectives from East Asia and Europe. 2007; 15–44. https://ink.library.smu.edu.sg/soe_research/596/

[35] George PP, Heng BH, De Castro Molina JA, Wong LY, Wei Lin NC, Cheah JT. Self-reported chronic diseases and health status and health service utilization—results from a community health survey in Singapore. Int J Equity Health. 2012; 11:44. 10.1186/1475-9276-11-44 22894180 PMC3490941

[36] DunnJR, HayesMV, HulchanskiJD, HwangSW, PotvinL. Housing as a socio-economic determinant of health: findings of a national needs, gaps and opportunities assessment.Can J Public Health. 2006 (Suppl 3); 97:S11–15, S12–17. 17357542

[37] Wang F, Zhen Q, Li K, Wen X. Association of socioeconomic status and health-related behavior with elderly health in China. PLoS One. 2018; 13:e0204237. 10.1371/journal.pone.0204237 30235282 PMC6147496

[38] Vasoo S, Lee J. Singapore: social development, housing and the Central Provident Fund. Int J Soc Welf. 2001; 10:276–83. 10.1111/1468-2397.00186

[39] Geronimus AT, Hicken M, Keene D, Bound J. "Weathering" and age patterns of allostatic load scores among blacks and whites in the United States. Am J Public Health. 2006; 96:826–33. 10.2105/AJPH.2004.060749 16380565 PMC1470581

[40] Bird CE, Seeman T, Escarce JJ, Basurto-Dávila R, Finch BK, Dubowitz T, Heron M, Hale L, Merkin SS, Weden M, Lurie N. Neighbourhood socioeconomic status and biological 'wear and tear' in a nationally representative sample of US adults. J Epidemiol Community Health. 2010; 64:860–65. 10.1136/jech.2008.084814 19759056 PMC3432399

[41] Kennedy BK, Berger SL, Brunet A, Campisi J, Cuervo AM, Epel ES, Franceschi C, Lithgow GJ, Morimoto RI, Pessin JE, Rando TA, Richardson A, Schadt EE, et al. Geroscience: linking aging to chronic disease. Cell. 2014; 159:709–13. 10.1016/j.cell.2014.10.039 25417146 PMC4852871

[42] Sierra F. The emergence of geroscience as an interdisciplinary approach to the enhancement of health span and life span. Cold Spring Harb Perspect Med. 2016; 6:a025163. 10.1101/cshperspect.a025163 26931460 PMC4817738

[43] Gruenewald TL, Karlamangla AS, Hu P, Stein-Merkin S, Crandall C, Koretz B, Seeman TE. History of socioeconomic disadvantage and allostatic load in later life. Soc Sci Med. 2012; 74:75–83. 10.1016/j.socscimed.2011.09.037 22115943 PMC3264490

[44] Horvath S. DNA methylation age of human tissues and cell types. Genome Biol. 2013; 14:R115. 10.1186/gb-2013-14-10-r115 24138928 PMC4015143

[45] Hannum G, Guinney J, Zhao L, Zhang L, Hughes G, Sadda S, Klotzle B, Bibikova M, Fan JB, Gao Y, Deconde R, Chen M, Rajapakse I, et al. Genome-wide methylation profiles reveal quantitative views of human aging rates. Mol Cell. 2013; 49:359–67. 10.1016/j.molcel.2012.10.016 23177740 PMC3780611

[46] Levine ME, Lu AT, Quach A, Chen BH, Assimes TL, Bandinelli S, Hou L, Baccarelli AA, Stewart JD, Li Y, Whitsel EA, Wilson JG, Reiner AP, et al. An epigenetic biomarker of aging for lifespan and healthspan. Aging (Albany NY). 2018; 10:573–91. 10.18632/aging.101414 29676998 PMC5940111

[47] Verschoor CP, Belsky DW, Ma J, Cohen AA, Griffith LE, Raina P. Comparing Biological Age Estimates Using Domain-Specific Measures From the Canadian Longitudinal Study on Aging. J Gerontol A Biol Sci Med Sci. 2021; 76:187–94. 10.1093/gerona/glaa151 32598446 PMC7812432

[48] Pyrkov TV, Slipensky K, Barg M, Kondrashin A, Zhurov B, Zenin A, Pyatnitskiy M, Menshikov L, Markov S, Fedichev PO. Extracting biological age from biomedical data via deep learning: too much of a good thing? Sci Rep. 2018; 8:5210. 10.1038/s41598-018-23534-9 29581467 PMC5980076

[49] Pyrkov TV, Fedichev PO. Biological age is a universal marker of aging, stress, and frailty. Biomarkers of Human Aging. 2019; 10:23–36. 10.1007/978-3-030-24970-0_3

[50] Pyrkov TV, Avchaciov K, Tarkhov AE, Menshikov LI, Gudkov AV, Fedichev PO. Longitudinal analysis of blood markers reveals progressive loss of resilience and predicts human lifespan limit. Nat Commun. 2021; 12:2765. 10.1038/s41467-021-23014-1 34035236 PMC8149842

[51] Carroll JE, Diez-Roux AV, Adler NE, Seeman TE. Socioeconomic factors and leukocyte telomere length in a multi-ethnic sample: findings from the multi-ethnic study of atherosclerosis (MESA). Brain Behav Immun. 2013; 28:108–14. 10.1016/j.bbi.2012.10.024 23142704 PMC3544984

[52] Woo J, Suen EW, Leung JC, Tang NL, Ebrahim S. Older men with higher self-rated socioeconomic status have shorter telomeres. Age Ageing. 2009; 38:553–58. 10.1093/ageing/afp098 19556325 PMC2729241

[53] Adams J, Martin-Ruiz C, Pearce MS, White M, Parker L, von Zglinicki T. No association between socio-economic status and white blood cell telomere length. Aging Cell. 2007; 6:125–28. 10.1111/j.1474-9726.2006.00258.x 17156082

[54] Geronimus AT. Deep integration: letting the epigenome out of the bottle without losing sight of the structural origins of population health. Am J Public Health. 2013 (Suppl 1); 103:S56–63. 10.2105/AJPH.2013.301380 23927509 PMC3786760

[55] Geronimus AT, Hicken MT, Pearson JA, Seashols SJ, Brown KL, Cruz TD. Do US Black Women Experience Stress-Related Accelerated Biological Aging?: A Novel Theory and First Population-Based Test of Black-White Differences in Telomere Length. Hum Nat. 2010; 21:19–38. 10.1007/s12110-010-9078-0 20436780 PMC2861506

[56] Sharfstein J, Sandel M, Kahn R, Bauchner H. Is child health at risk while families wait for housing vouchers? Am J Public Health. 2001; 91:1191–92. 10.2105/ajph.91.8.1191 11499101 PMC1446743

[57] Cizza G, Brady LS, Calogero AE, Bagdy G, Lynn AB, Kling MA, Blackman MR, Chrousos GP, Gold PW. Central hypothyroidism is associated with advanced age in male Fischer 344/N rats: *in vivo* and *in vitro* studies. Endocrinology. 1992; 131:2672–80. 10.1210/endo.131.6.1446609 1446609

[58] Gan DRY, Fung JC, Cho IS. Neighborhood atmosphere modifies the eudaimonic impact of cohesion and friendship among older adults: A multilevel mixed-methods study. Soc Sci Med. 2021; 270:113682. 10.1016/j.socscimed.2021.113682 33461036

[59] Gan DRY, Chaudhury H, Mann J, Wister AV. Dementia-friendly neighbourhood and the built environment: A scoping review. Gerontologist. 2021. [Epub ahead of print]. 10.1093/geront/gnab019 33564829

[60] Pearlin LI, Lieberman MA. Social sources of emotional distress. Res Community Ment Health. 1979; 1:217–48.

[61] Pearlin LI, Johnson JS. Marital status, life-strains and depression. Am Sociol Rev. 1977; 42:704–15. 10.2307/2094860 931191

[62] Thoits PA. Life stress, social support, and psychological vulnerability: epidemiological considerations. J Community Psychol. 1982; 10:341–62. 10.1002/1520-6629(198210)10:4<341::aid-jcop2290100406>3.0.co;2-j 10298894

[63] Afzal S, Tybjærg-Hansen A, Jensen GB, Nordestgaard BG. Change in Body Mass Index Associated With Lowest Mortality in Denmark, 1976-2013. JAMA. 2016; 315:1989–96. 10.1001/jama.2016.4666 27163987

[64] Dahl AK, Fauth EB, Ernsth-Bravell M, Hassing LB, Ram N, Gerstof D. Body mass index, change in body mass index, and survival in old and very old persons. J Am Geriatr Soc. 2013; 61:512–18. 10.1111/jgs.12158 23452127 PMC3628079

[65] Ng TKS, Slowey PD, Beltran D, Ho RCM, Kua EH, Mahendran R. Effect of mindfulness intervention versus health education program on salivary Aβ-42 levels in community-dwelling older adults with mild cognitive impairment: A randomized controlled trial. J Psychiatr Res. 2021; 136:619–25. 10.1016/j.jpsychires.2020.10.038 33199051

[66] Gelman A. The problems with *p*-values are not just with *p*-values. The American Statistician. 2016; 70:1.

[67] Ng TKS, Fam J, Feng L, Cheah IK, Tan CT, Nur F, Wee ST, Goh LG, Chow WL, Ho RC, Kua EH, Larbi A, Mahendran R. Mindfulness improves inflammatory biomarker levels in older adults with mild cognitive impairment: a randomized controlled trial. Transl Psychiatry. 2020; 10:21. 10.1038/s41398-020-0696-y 32066726 PMC7026149

[68] Ng TKS, Matchar DB, Sultana R, Chan A. Effects of Self-Care for Older PErsons (SCOPE) on Functional and Physiological Measures: A Cluster Randomized Controlled Trial. J Clin Med. 2020; 9:885. 10.3390/jcm9030885 32213860 PMC7141527

[69] Pyrkov TV, Sokolov IS, Fedichev PO. Deep longitudinal phenotyping of wearable sensor data reveals independent markers of longevity, stress, and resilience. Aging (Albany NY). 2021; 13:7900–13. 10.18632/aging.202816 33735108 PMC8034931

[70] Pyrkov TV, Getmantsev E, Zhurov B, Avchaciov K, Pyatnitskiy M, Menshikov L, Khodova K, Gudkov AV, Fedichev PO. Quantitative characterization of biological age and frailty based on locomotor activity records. Aging (Albany NY). 2018; 10:2973–90. 10.18632/aging.101603 30362959 PMC6224248

[71] Bryant T. The current state of housing in Canada as a social determinant of health. Policy Options-Montreal. The Institute for Research on Public Policy. 2003; 24:52–56.

[72] Chan A, Matchar DB, Tsao MA, Harding S, Chiu CT, Tay B, Raman P, Pietryla Z, Klein MK, Haldane VE. Self-Care for Older People (SCOPE): a cluster randomized controlled trial of self-care training and health outcomes in low-income elderly in Singapore. Contemp Clin Trials. 2015; 41:313–24. 10.1016/j.cct.2015.01.001 25583272

[73] Ng TKS, Kovalik JP, Ching J, Chan AW, Matchar DB. Novel metabolomics markers are associated with pre-clinical decline in hand grip strength in community-dwelling older adults. Mech Ageing Dev. 2021; 193:111405. 10.1016/j.mad.2020.111405 33217429

[74] Zenin A, Tsepilov Y, Sharapov S, Getmantsev E, Menshikov LI, Fedichev PO, Aulchenko Y. Identification of 12 genetic loci associated with human healthspan. Commun Biol. 2019; 2:41. 10.1038/s42003-019-0290-0 30729179 PMC6353874

[75] Green MS, Symons MJ. A comparison of the logistic risk function and the proportional hazards model in prospective epidemiologic studies. J Chronic Dis. 1983; 36:715–23. 10.1016/0021-9681(83)90165-0 6630407

[76] Abbott RD. Logistic regression in survival analysis. Am J Epidemiol. 1985; 121:465–71. 10.1093/oxfordjournals.aje.a114019 4014135

[77] Ganna A, Ingelsson E. 5 year mortality predictors in 498,103 UK Biobank participants: a prospective population-based study. Lancet. 2015; 386:533–40. 10.1016/S0140-6736(15)60175-1 26049253

