# Supplementary material for: Association between housing type and accelerated biological aging in different sexes: moderating effects of health behaviors
Source: Aging (Albany NY). 2021 Aug 29;13(16):20029–49. doi: 10.18632/aging.203447 (PMC8436907; doi:10.18632/aging.203447)
Supplement: Supplementary Materials [file aging-13-203447-s001.pdf]

SUPPLEMENTARY MATERIALS

Supplementary Materials

Study participants

In the main study, we recruited research participants from 14 Senior Activity Centers (SACs) which were located in low-SES areas, selected from a total of 42 in Singapore. Subsequently, from a list of individuals attached to each SAC, we randomly selected potential participants using a computerized random number generator. Participants were included if they were aged 55 years and above, affiliated with one of the SACs, had consented to future data use and blood draws. Participants were excluded if they had cognitive impairment as indicated by Abbreviated Mental Test (AMT) score < 5, had a debilitating or terminal illness, or activities of daily living (ADL) disability that rendered them physically unfit to participate at the time of screening. Participants were also excluded if they were on either chemotherapy or dialysis or received medications for major psychiatric disorders at the time.

Covariates

Ethnicity

Based on the distribution of our sample, we categorized ethnicity into two groups, namely Chinese and non-Chinese, which comprised the Malay and Indian.

Marital status

We categorized marital status into two groups, namely married and not married, which included those who were single, widowed, and divorced.

Physical and social activity levels

We asked three questions for each category of the activities. For physical activities, we asked how much total time the participants spent on each of the following activities: 1) performing stretching or strengthening exercises, 2) walked for exercise purposes, and 3) performing vigorous exercise. For social activities, we asked how often the participants performed any of the following in the past week: 1) attended community or neighbourhood event, 2) went out to eat, and 3) attended church, synagogue, mosque, temple or other places of worship. For physical activities, participants who performed one of the activities at least 30–60 minutes per week were given 1 point. For social activities, participants who performed one of the activities at least once a week were given 1 point. The total scores for each of the two types of activities were calculated. The total scores ranged from 0 to 3, with a higher score representing a higher level of activities.
