# Supplementary material for: Association between housing type and accelerated biological aging in different sexes: moderating effects of health behaviors
Source: Aging (Albany NY). 2021 Aug 29;13(16):20029–49. doi: 10.18632/aging.203447 (PMC8436907; doi:10.18632/aging.203447)
Supplement: Supplementary Tables [file aging-13-203447-s002.pdf]

SUPPLEMENTARY TABLES

Supplementary Table 1A. ***Bivariate*** **associations between each SES indicator with biological age and biological age acceleration – Total sample.**

Indicators of SES	Biological age; *r* (*P* value)	Biological age acceleration; *r* (*P* value)
Housing type	**0.191 (<0.001***)**	**0.127 (0.007**)**
Education level	**0.104 (0.022*)**	0.013 (0.402)
Longest occupational role	−0.059 (0.126)	−0.079 (0.063)
Income level	−0.008 (0.444)	−0.002 (0.484)
Perceived income adequacy	−0.042 (0.209)	−0.014 (0.392)

Supplementary Table 1B. ***Bivariate*** **associations between each SES indicator with biological age and biological age acceleration – Men.**

Indicators of SES	Biological age; *r* (*P* value)	Biological age acceleration; *r* (*P* value)
Housing type	0.117 (0.095)	0.138 (0.061)
Education level	0.140 (0.059)	0.081 (0.182)
Longest occupational role	**−0.181 (0.021*)**	**−0.173 (0.026*)**
Income level	**0.152 (0.048*)**	**0.153 (0.047*)**
Perceived income adequacy	−0.124 (0.083)	−0.079 (0.190)

Supplementary Table 1C. ***Bivariate*** **associations between each SES indicator with biological age and biological age acceleration – Women.**

Indicators of SES	Biological age; *r* (*P* value)	Biological age acceleration; *r* (*P* value)
Housing type	**0.183 (0.002**)**	**0.135 (0.017*)**
Education level	**0.122 (0.027*)**	−0.023 (0.359)
Longest occupational role	−0.034 (0.299)	−0.005 (0.468)
Income level	−0.085 (0.099)	−0.073 (0.135)
Perceived income adequacy	−0.027 (0.334)	0.018 (0.388)

Supplementary Table 2A. Associations between housing type and biological age/biological age acceleration – Total sample – Extension of Table 2 with all covariates and standardized β presented.

Models		Biological age			Biological age acceleration		
		β (95% CI)	*Standardized* β	*P* value	β (95% CI)	*Standardized* β	*P* value
**Total Sample**							
1	Housing Type	0.263 (0.12 to 0.406)	0.191	**<0.001*** **	0.156 (0.027 to 0.285)	0.127	**0.018***
2	Housing Type	0.173 (0.024 to 0.323)	0.126	**0.023***	0.166 (0.028 to 0.305)	0.135	**0.019***
	chronological age	0.017 (0.009 to 0.025)	0.219	**<0.001*** **	0 (−0.007 to 0.008)	0.001	0.98
	sex	−0.12 (−0.259 to 0.019)	−0.09	0.091	0.06 (−0.069 to 0.189)	0.05	0.361
	ethnicity	−0.03 (−0.206 to 0.145)	−0.018	0.733	−0.025 (−0.187 to 0.138)	−0.016	0.767
3	Housing Type	0.199 (0.038 to 0.36)	0.145	**0.015***	0.183 (0.033 to 0.334)	0.149	**0.017***
	chronological age	0.016 (0.008 to 0.025)	0.207	**<0.001*** **	0 (−0.008 to 0.009)	0.005	0.936
	sex	−0.154 (−0.299 to −0.01)	−0.116	**0.037***	0.041 (−0.093 to 0.176)	0.035	0.546
	ethnicity	−0.032 (−0.218 to 0.155)	−0.019	0.738	−0.012 (−0.186 to 0.162)	−0.008	0.893
	marital status	0.012 (−0.124 to 0.148)	0.009	0.863	0.022 (−0.105 to 0.149)	0.019	0.736
	education level	0.038 (−0.109 to 0.186)	0.03	0.609	−0.018 (−0.155 to 0.12)	−0.016	0.8
	longest occupational role	−0.157 (−0.388 to 0.074)	−0.074	0.182	−0.135 (−0.35 to 0.081)	−0.071	0.22
	income level	−0.086 (−0.24 to 0.067)	−0.06	0.269	−0.035 (−0.178 to 0.109)	−0.027	0.636
	perceived income adequacy	−0.064 (−0.204 to 0.077)	−0.049	0.372	−0.031 (−0.162 to 0.1)	−0.027	0.644
4	Housing Type	0.193 (0.032 to 0.354)	0.14	**0.019***	0.18 (0.029 to 0.331)	0.146	**0.02***
	chronological age	0.017 (0.008 to 0.026)	0.218	**<0.001*** **	0.001 (−0.008 to 0.009)	0.01	0.867
	sex	−0.204 (−0.357 to −0.052)	−0.154	**0.009****	0.016 (−0.127 to 0.159)	0.014	0.825
	ethnicity	0.042 (−0.152 to 0.237)	0.025	0.668	0.026 (−0.157 to 0.208)	0.017	0.782
	marital status	−0.008 (−0.144 to 0.129)	−0.006	0.909	0.011 (−0.117 to 0.139)	0.01	0.863
	education level	0.017 (−0.132 to 0.166)	0.013	0.822	−0.03 (−0.169 to 0.11)	−0.026	0.676
	longest occupational role	−0.162 (−0.392 to 0.069)	−0.076	0.169	−0.136 (−0.352 to 0.081)	−0.072	0.219
	income level	−0.112 (−0.267 to 0.043)	−0.077	0.155	−0.048 (−0.193 to 0.097)	−0.037	0.518
	perceived income adequacy	−0.057 (−0.199 to 0.084)	−0.044	0.427	−0.027 (−0.16 to 0.106)	−0.023	0.692
	BMI	0.016 (0.001 to 0.031)	0.121	**0.032***	0.008 (−0.006 to 0.022)	0.068	0.25
	smoking status	−0.073 (−0.31 to 0.163)	−0.034	0.542	−0.03 (−0.253 to 0.192)	−0.016	0.789
	alcohol drinker	−0.081 (−0.282 to 0.121)	−0.043	0.432	−0.053 (−0.242 to 0.137)	−0.032	0.585
5	Housing Type	0.189 (0.027 to 0.35)	0.137	**0.022***	0.178 (0.027 to 0.33)	0.145	**0.021***
	chronological age	0.017 (0.008 to 0.026)	0.219	**<0.001*** **	0.001 (−0.008 to 0.009)	0.012	0.847
	sex	−0.204 (−0.358 to −0.051)	−0.153	**0.009****	0.014 (−0.13 to 0.157)	0.011	0.853
	ethnicity	0.027 (−0.168 to 0.222)	0.016	0.786	0.012 (−0.171 to 0.195)	0.008	0.895
	marital status	−0.014 (−0.151 to 0.123)	−0.011	0.837	0.007 (−0.122 to 0.135)	0.006	0.92
	education level	0.02 (−0.128 to 0.168)	0.016	0.792	−0.026 (−0.165 to 0.113)	−0.023	0.716
	longest occupational role	−0.18 (−0.412 to 0.051)	−0.085	0.126	−0.157 (−0.373 to 0.06)	−0.083	0.155
	income level	−0.114 (−0.269 to 0.041)	−0.079	0.148	−0.052 (−0.197 to 0.093)	−0.04	0.481
	perceived income adequacy	−0.068 (−0.21 to 0.074)	−0.053	0.347	−0.039 (−0.172 to 0.094)	−0.033	0.568
	BMI	0.017 (0.002 to 0.031)	0.127	**0.025***	0.009 (−0.005 to 0.023)	0.075	0.201
	smoking status	−0.058 (−0.295 to 0.179)	−0.027	0.63	−0.015 (−0.237 to 0.207)	−0.008	0.894
	alcohol drinker	−0.083 (−0.284 to 0.118)	−0.044	0.416	−0.055 (−0.244 to 0.133)	−0.033	0.563
	social activity levels	−0.091 (−0.179 to −0.002)	−0.108	**0.046***	−0.092 (−0.176 to −0.009)	−0.123	**0.03***
	physical activity levels	0.031 (−0.043 to 0.105)	0.045	0.407	0.041 (−0.028 to 0.111)	0.067	0.243

*indicates $p < 0.05$, **indicates p<0.01, and ***indicates $p < 0.001$. Abbreviations: 95% CI: 95% confidence interval; BMI: body-mass index.

Supplementary Table 2B. Associations between housing type and biological age/biological age acceleration – Men subgroup analyses – Extension of Table 2 with all covariates and standardized β presented.

Models		Biological Age			Biological Age Acceleration		
		β (95% CI)	*Standardized* β	*P* value	β (95% CI)	*Standardized* β	*P* value
**Total sample**							
1	Housing Type	0.188 (−0.104 to 0.479)	0.117	0.204	0.202 (−0.064 to 0.467)	0.138	0.135
2	Housing Type	0.148 (−0.144 to 0.44)	0.092	0.318	0.192 (−0.079 to 0.463)	0.131	0.163
	chronological age	0.014 (0 to 0.027)	0.188	**0.043***	0.001 (−0.011 to 0.013)	0.019	0.837
	ethnicity	−0.046 (−0.326 to 0.234)	−0.03	0.746	−0.048 (−0.307 to 0.212)	−0.034	0.716
3	Housing Type	0.101 (−0.201 to 0.404)	0.063	0.508	0.154 (−0.13 to 0.438)	0.105	0.285
	chronological age	0.015 (0.001 to 0.028)	0.203	**0.035***	0.002 (−0.01 to 0.015)	0.034	0.725
	ethnicity	−0.09 (−0.385 to 0.205)	−0.058	0.548	−0.085 (−0.361 to 0.192)	−0.06	0.544
	marital status	−0.042 (−0.264 to 0.179)	−0.037	0.705	−0.022 (−0.23 to 0.185)	−0.021	0.832
	education level	0.033 (−0.19 to 0.257)	0.029	0.768	0.001 (−0.209 to 0.211)	0.001	0.993
	longest occupational role	−0.236 (−0.515 to 0.042)	−0.165	0.095	−0.189 (−0.45 to 0.072)	−0.144	0.153
	income level	0.182 (−0.078 to 0.442)	0.136	0.168	0.175 (−0.069 to 0.418)	0.143	0.158
	perceived income adequacy	−0.102 (−0.33 to 0.125)	−0.089	0.374	−0.077 (−0.29 to 0.136)	−0.074	0.475
4	Housing Type	0.109 (−0.188 to 0.405)	0.068	0.468	0.155 (−0.126 to 0.436)	0.106	0.277
	chronological age	0.016 (0.003 to 0.029)	0.222	**0.02***	0.004 (−0.009 to 0.016)	0.056	0.567
	ethnicity	−0.016 (−0.308 to 0.276)	−0.01	0.913	−0.031 (−0.308 to 0.247)	−0.022	0.827
	marital status	−0.053 (−0.269 to 0.162)	−0.047	0.623	−0.033 (−0.237 to 0.171)	−0.032	0.749
	education level	0.049 (−0.171 to 0.269)	0.043	0.658	0.013 (−0.196 to 0.222)	0.012	0.904
	longest occupational role	−0.271 (−0.542 to 0)	−0.189	0.05	−0.214 (−0.472 to 0.043)	−0.163	0.102
	income level	0.106 (−0.15 to 0.362)	0.079	0.413	0.116 (−0.128 to 0.359)	0.095	0.348
	perceived income adequacy	−0.042 (−0.267 to 0.184)	−0.037	0.714	−0.031 (−0.245 to 0.183)	−0.03	0.775
	BMI	0.039 (0.012 to 0.066)	0.26	**0.006***	0.033 (0.007 to 0.059)	0.242	**0.013***
	smoking status	−0.102 (−0.363 to 0.159)	−0.073	0.442	−0.048 (−0.296 to 0.2)	−0.038	0.701
	alcohol drinker	0.049 (−0.195 to 0.292)	0.036	0.693	0.036 (−0.195 to 0.267)	0.029	0.756
5	Housing Type	0.108 (−0.189 to 0.405)	0.068	0.471	0.154 (−0.127 to 0.436)	0.105	0.28
	chronological age	0.016 (0.002 to 0.029)	0.219	**0.022***	0.003 (−0.009 to 0.016)	0.051	0.601
	ethnicity	−0.05 (−0.349 to 0.248)	−0.033	0.739	−0.055 (−0.338 to 0.228)	−0.039	0.702
	marital status	−0.057 (−0.276 to 0.161)	−0.05	0.605	−0.026 (−0.233 to 0.181)	−0.025	0.802
	education level	0.038 (−0.183 to 0.259)	0.033	0.734	0.005 (−0.205 to 0.215)	0.005	0.964
	longest occupational role	−0.282 (−0.554 to −0.009)	−0.196	**0.043***	−0.222 (−0.48 to 0.037)	−0.169	0.092
	income level	0.106 (−0.152 to 0.364)	0.079	0.416	0.109 (−0.136 to 0.353)	0.089	0.381
	perceived income adequacy	−0.083 (−0.32 to 0.154)	−0.072	0.49	−0.078 (−0.303 to 0.147)	−0.075	0.492
	BMI	0.039 (0.012 to 0.067)	0.263	**0.005***	0.033 (0.007 to 0.059)	0.242	**0.013***
	smoking status	−0.094 (−0.355 to 0.168)	−0.067	0.479	−0.042 (−0.29 to 0.206)	−0.033	0.737
	alcohol drinker	0.04 (−0.205 to 0.285)	0.029	0.748	0.034 (−0.199 to 0.266)	0.027	0.775
	social activity levels	−0.089 (−0.233 to 0.055)	−0.117	0.221	−0.071 (−0.207 to 0.065)	−0.102	0.304
	physical activity levels	0.041 (−0.085 to 0.166)	0.061	0.523	0.069 (−0.05 to 0.188)	0.113	0.252

*indicates $p < 0.05$, **indicates $p < 0.01$, and ***indicates $p < 0.001$. Abbreviations: 95% CI: 95% confidence interval; BMI: body-mass index.

Supplementary Table 2C. Associations between housing type and biological age/biological age acceleration – Women subgroup analyses – Extension of Table 2 with all covariates and standardized β presented.

Models		Biological Age			Biological Age Acceleration		
		β (95% CI)	*Standardized* β	*P* value	β (95% CI)	*Standardized* β	*P* value
**Total sample**							
1	Housing Type	0.246 (0.073 to 0.419)	0.183	**0.006****	0.162 (0.006 to 0.319)	0.135	**0.042***
2	Housing Type	0.176 (−0.003 to 0.355)	0.131	0.054	0.163 (−0.004 to 0.329)	0.135	0.055
	chronological age	0.019 (0.009 to 0.03)	0.236	**<0.001*****	−0.001 (−0.01 to 0.009)	−0.009	0.898
	ethnicity	−0.032 (−0.261 to 0.196)	−0.019	0.782	−0.009 (−0.222 to 0.203)	−0.006	0.932
3	Housing Type	0.256 (0.062 to 0.45)	0.191	**0.01****	0.218 (0.036 to 0.4)	0.181	**0.019***
	chronological age	0.02 (0.009 to 0.032)	0.252	**0.001*****	0.002 (−0.009 to 0.013)	0.024	0.759
	ethnicity	−0.017 (−0.26 to 0.227)	−0.01	0.893	0.027 (−0.201 to 0.255)	0.018	0.814
	marital status	0.091 (−0.085 to 0.267)	0.069	0.309	0.077 (−0.089 to 0.242)	0.065	0.362
	education level	0 (−0.201 to 0.201)	0	0.999	−0.058 (−0.247 to 0.13)	−0.049	0.542
	longest occupational role	0.037 (−0.365 to 0.439)	0.012	0.858	−0.011 (−0.387 to 0.366)	−0.004	0.954
	income level	−0.229 (−0.424 to −0.035)	−0.155	**0.021***	−0.145 (−0.328 to 0.037)	−0.109	0.118
	perceived income adequacy	−0.03 (−0.21 to 0.149)	−0.022	0.74	0.007 (−0.162 to 0.175)	0.005	0.939
4	Housing Type	0.259 (0.065 to 0.452)	0.193	**0.009****	0.221 (0.039 to 0.403)	0.183	**0.018***
	chronological age	0.02 (0.008 to 0.032)	0.247	**0.001****	0.001 (−0.01 to 0.012)	0.014	0.857
	ethnicity	0.048 (−0.211 to 0.308)	0.028	0.714	0.038 (−0.206 to 0.282)	0.024	0.76
	marital status	0.079 (−0.099 to 0.256)	0.06	0.383	0.079 (−0.087 to 0.246)	0.067	0.349
	education level	−0.036 (−0.239 to 0.166)	−0.027	0.723	−0.076 (−0.267 to 0.115)	−0.063	0.436
	longest occupational role	0.081 (−0.322 to 0.484)	0.027	0.692	0.02 (−0.359 to 0.4)	0.008	0.915
	income level	−0.273 (−0.47 to −0.075)	−0.184	**0.007****	−0.169 (−0.355 to 0.017)	−0.128	0.074
	perceived income adequacy	−0.01 (−0.192 to 0.172)	−0.007	0.913	0.026 (−0.145 to 0.197)	0.021	0.763
	BMI	0.008 (−0.01 to 0.025)	0.06	0.397	0 (−0.017 to 0.016)	−0.004	0.956
	smoking status	0.23 (−0.239 to 0.699)	0.063	0.334	0.275 (−0.167 to 0.717)	0.083	0.221
	alcohol drinker	−0.361 (−0.683 to −0.039)	−0.151	**0.028****	−0.251 (−0.555 to 0.052)	−0.117	0.104
5	Housing Type	0.249 (0.054 to 0.444)	0.185	**0.013***	0.209 (0.026 to 0.392)	0.173	**0.026***
	chronological age	0.02 (0.008 to 0.032)	0.248	**0.001****	0.001 (−0.01 to 0.012)	0.016	0.841
	ethnicity	0.032 (−0.229 to 0.294)	0.019	0.807	0.019 (−0.226 to 0.264)	0.012	0.877
	marital status	0.073 (−0.105 to 0.25)	0.055	0.421	0.072 (−0.094 to 0.239)	0.061	0.393
	education level	−0.029 (−0.232 to 0.174)	−0.022	0.779	−0.067 (−0.257 to 0.123)	−0.056	0.489
	longest occupational role	0.066 (−0.338 to 0.471)	0.022	0.746	0.004 (−0.376 to 0.384)	0.001	0.984
	income level	−0.27 (−0.469 to −0.072)	−0.183	**0.008****	−0.166 (−0.352 to 0.019)	−0.125	0.079
	perceived income adequacy	−0.008 (−0.189 to 0.173)	−0.006	0.933	0.029 (−0.141 to 0.199)	0.024	0.739
	BMI	0.008 (−0.01 to 0.026)	0.063	0.371	<0.001 (−0.017 to 0.017)	<0.001	0.995
	smoking status	0.285 (−0.187 to 0.757)	0.077	0.235	0.339 (−0.104 to 0.782)	0.103	0.133
	alcohol drinker	−0.354 (−0.676 to −0.032)	−0.148	**0.031***	−0.243 (−0.545 to 0.059)	−0.113	0.114
	social activity levels	−0.101 (−0.214 to 0.012)	−0.116	0.081	−0.117 (−0.223 to −0.011)	−0.15	**0.031***
	physical activity levels	0.021 (−0.071 to 0.114)	0.031	0.651	0.024 (−0.063 to 0.111)	0.038	0.59

*indicates $p < 0.05$, **indicates $p < 0.01$, and ***indicates $p < 0.001$. Abbreviations: 95% CI: 95% confidence interval; BMI: body-mass index.
